# On the Treatment of Dysentery

**Published:** 1865-08

**Authors:** D. B. Trimble

**Affiliations:** Chicago, Ill.


CHICAGO

MEDICAL JOURNAL.

VOL. XXII.] AUGUST, 1865. [No. 8.

### ORIGINAL COMMUNICATIONS.

### ON THE TREATMENT OF DYSENTERY.

By Dr. D. B. TRIMBLE, of Chicago, Ill.

As the season is approaching when dysentery is generally more or less prevalent, perhaps a few remarks on the treatment of this disease, from one who has had considerable experience with it, may not be unacceptable to the younger readers of the JOURNAL. I am aware that the subject has been very ably, extensively and almost exhaustively treated; nor do I propose to suggest any novel mode of treatment, but simply to state what, in my hand, has been followed by satisfactory results.

As dysentery arises from a variety of causes, and is frequently variously complicated, it is obvious that no exclusive or single mode of treatment (as has been recommended by some authorities) is applicable to every case; but, to be successful, must be varied according to the symptoms, causes, age and constitution of the patient, and to the concomitant diseases, if any are present.

The epidemic form of dysentery, or rather that produced by epidemic influence, (for I do not know that there is any essential difference in the symptoms, except their greater severity, between the epidemic and other varieties), is the one I shall have chiefly in view in the following remarks.

The first summer that I practiced medicine, (in an agricultural community on the eastern shore of Maryland), the disease prevailed very extensively and in an aggravated form, few families escaping altogether, and in some families all, or nearly all, the members being affected. The symptoms were then—as I have always met with them since, to a greater or less extent in the disease—a frequent and urgent inclination to evacuate the bowels; the discharges being generally composed of mucus of a whitish, coagulated appearance, frequently mixed with blood, and sometimes with bile; sometimes (but generally before I would be called to the case) preceeded by feculent or bilious evacuations. Those cases in which a mucous character of discharges prevailed extensively, or exclusively, were generally the most painful and unmanageable, and I was soon led to look upon a free evacuation of blood in the early stages as a favorable symptom. Accompanying this character of the discharges, there would be tormina and tenesmus, more or less fever, thirst, nausea often, sometimes vomiting, pain in the back, scanty urine, and a dry skin. There was often tenderness of the abdomen, but seldom tumefaction.

In regard to therapeutics, venesection was seldom resorted to; and the few instances in which I did bleed were in persons of good constitutions, in the vigor of life, and where the fever ran high, and in these cases with much benefit. Let me here remark that in regard to blood-letting, I think the tendency, or *fashion*, (for there is a fashion in therapeutics, as in most other things), is as much to its improper neglect at the present day, as it was too freely resorted to by the physicians of half or even a quarter of a century ago.

At the time of the epidemic under consideration, an excellent physician, then about eighteen years in practice, lived a few miles east of me, and another, who had been a practitioner thirty five or forty years, a few miles to the west. Those gentlemen bled freely, and were very unfortunate in the results of their treatment I remember one family, after having lost two of its members under the depleting mode, in the third case sent for me. I did *not* bleed, and the patient recovered, though apparently as ill as the others. Though there are diseases in which I would resort to venesection, where many physicians of the present day would *not*, yet dysentery is not one of those diseases, except in peculiar circumstances. The disease often produces great debility, and is sometimes prolonged to several weeks, so that it is necessary to economize the strength of the system as much as circumstances will permit; and unless the inflammation of the colon and rectum is of a very active grade, we can generally succeed without it.

From the absence of the bile in the dejections, it was evident that the liver was in a torpid condition, and therefore an agent that would unlock it and enable it to eliminate its secretions was indicated. What could fulfill this indication better than mercury? I therefore generally commenced my treatment with a dose of calomel—eight or ten grains to an adult,—followed, in four or five hours, by one-half oz. or one oz. Ol. Ricini, if the stomach was not irritable, or the fever not very high; but, if this was the case, a saline; and in the event of nausea, an effervescing aperient was substituted. This almost invariably alleviated the sufferings, and frequently was sufficient to effect a cure. But if the disease was not conquered, then I continued the calomel in diminished doses: two grains once in two, three or four hours, combined with one-half or one-quarter grain of opium, and one to two grains of ipecacuanha, with a view of alleviating the tenesmus and exciting the action of the skin, which, like the liver, is in a torpid state. This plan would be continued for twenty-four or forty-eight hours, when the castor oil would be repeated. When this had produced a free secretion of bile and of perspiration, in the great majority of cases, a cure would be accomplished; but sometimes an irritable state of the bowels would continue after the constitutional symptoms had subsided, as would be indicated by some tenesmus and inclination to stool, with very little discharge. In these cases, I continued the opium and ipecac, omiting the calomel, and substituting, sometimes, one or two grains of plumb. acet. to each dose. The local remedies generally of enemata of some mulcilage, as thin clear starch, in small quantities—about a gill—combined with thirty to sixty drops of laudanum, or its equivalent of sulph. of morphia two, three or four times a day. This was an excellent adjuvant in soothing the bowels by alleviating the tenesmus. I directed small injections, as they caused less irritation, were more likely to be retained, and therefore accomplished the object more perfectly. Warm anodyne, or stimulating embrocations to the abdomen, demulcent drinks, and a bland liquid diet, together with perfect rest, a pure atmosphere, and cleanliness, generally accomplished a cure. In convalescence, I seldom found it necessary to give tonics, or stimulants, though occasionally I had to resort to the latter in cases of great prostration.

During this, my first experience with dysentery, I treated just one hundred cases. Of this number, ten, or just ten per cent., died.

A singular circumstance occurred during the prevalence of this epidemic, which I have frequently noticed since, viz: that the negro race succumb to severe diseases much more readily than the whites. Of those I treated at that time, ten were colored persons; and although they had as much attention as my other patients, and as many comforts and facilities for recovery as many of the whites, yet of this number, five, or fifty per cent., died; while of the ninety whites treated, but five, or five and six-tenths per cent., died. But among the deaths of the blacks, two were boys, brothers, ten and eleven years old, who had been much emaciated, but were progressing towards health, when their parents yielded to their desire for *green apples*, (which hung, in their view, on a tree by the door,) and after partaking freely of them, they had a relapse, and in a few days died.

Another epidemic—in 1851—occurred while I was in Burlington, New Jersey, and I saw no reason to modify the treatment used in the former epidemic, except that I do not recollect having bled in any case, having a much more luxurious and delicate class of patients to treat than formerly. I have known it to prevail epidemically in other years, but not so extensively, and never knew a year to pass without witnessing more or less of it.

I know that the remedies for dysentery are almost innumerable, and some are said to be unfailing; but I must be convinced of the rationale of their operation before I can have undoubting faith in their curative powers, even when endorsed by the high and respectable names that sometimes accompany them. This I have not yet understood. Such are opium, very large doses of ipecacuanha, neutral salts, combined with mineral acids, etc. If my idea of the proximate causes of the disease is correct, I do not comprehend how these modes of treatment can have such speedy and universally successful terminations. That the purgative and depleting effects of the salts would fulfill one indication, the sedative effects of the opium another, and the diapheretic and alterative effect of the ipecac, a third, there can be no doubt; but to treat severe cases by either of these modes exclusively, I should be reluctant to do, for none of them, singly, can fulfill all the indications, or produce all the effects required in the treatment of dysentery. Opium, though an indispensible agent in severe cases, I have seen used to a dangerous, if not fatal, extent, when almost relied on. Ipecacuanha is almost as essential, but Icannot see the necessity of giving it in such enormous doses—one to two drahms—as a diphoretic, and as such is it chiefly, if not altogether, beneficial.

When dysentery arises in malarial districts, (where it is very apt to), and especially if it takes an intermittent or remittent form, the administration of quinia, in addition to the treatment already recommended, will be found very useful. Where the disease merges into the chronic form, I have found the Ol. Terebinth. and astringent enemata very beneficial.

